# Effect of Fatty Acyl Composition for Lysophosphatidylinositol on Neuroinflammatory Responses in Primary Neuronal Cultures

**DOI:** 10.21203/rs.3.rs-5742954/v1

**Published:** 2025-01-08

**Authors:** Douglas E. Brenneman, Dean Petkanas, Michael Ippolito, Sara Jane Ward

**Affiliations:** Kannalife Sciences, Inc Pennsylvania Biotechnology Center; Kannalife Sciences, Inc Pennsylvania Biotechnology Center; Temple University; Temple University

**Keywords:** lysophosphatidylinositol, neuroinflammation, cytokines, hippocampus, GPR55, dorsal root ganglion

## Abstract

Lysophosphatidylinositol (LPI) is an endogenous signaling molecule for the GPR55 receptor. Previous studies have shown that arachidonoyl-lysophosphatidylinositol (LPI-20:4) produced an increase in the inflammatory mediators NLPR3 (inflammasome – 3 marker) and IL-1b in neurons from both rat dorsal root ganglion (DRG) and hippocampal cultures. Because LPI is comprised of a family of lipid structures that vary in fatty acyl composition, the current work examined neuroinflammatory responses to various LPI structures in DRG and hippocampal cultures as assessed by high content fluorescent imaging. Major endogenous LPI fatty acyl structures consisting of 16:0, 18:0, 18:1 or 20:4 were compared for their effects on IL-1b, NLRP3 and GPR55 immunoreactive areas of neurites and cell bodies after a 6-hour treatment. Among these four LPI structures, only LPI-20:4 treatment produced increases in immunoreactive areas for GPR55, NLRP3 and IL-1b in DRG and hippocampal neurites. In contrast, all other LPI structures tested produced a decrease in all of these inflammatory immunoreactive areas in both neurites and cell bodies. Additional studies with LPI-20:4 treatment indicated that IL-6, IL-18 and TNF-a were significantly increased in neurites of DRG and hippocampal cultures. However, oleoyl-lysophosphatidylinositol (LPI-18:1) treatment produced decreases in these three cytokines. Using the viability dye alamar blue, LPI-20:4 was shown to produce concentration-dependent decreases, whereas all other endogenous LPI structures produced increases with this assay. These studies indicate that fatty acyl structure is the major determinant of LPI for neuroinflammatory responses in DRG and hippocampal cultures, with LPI-20:4 showing pro-inflammatory effects and all other endogenous LPIs tested exhibited anti-inflammatory responses.

## Introduction

Neuroinflammation is widely recognized as one of the major challenges to the homeostatic control of the nervous system. Chronic imbalances of neuroinflammatory processes may result in catastrophic consequences with the eventual emergence of neuronal damage or loss accompanied by irreversible impairment of neurological and cognitive function. Among the pathological disorders of the nervous system that have been associated with neuroinflammatory components are Alzheimer’s disease (Media-Ver et al., 2020), Parkinson’s disease (Pajares et al., 2020), depression (Troubat et al., 2020) and peripheral neuropathy (Fumagalli et al., 2021). While imbalances in the homeostatic control of neuroinflammation are widespread, the identification and recognition of the roles of important molecular mediators of neuroinflammation are daunting due to the known complexities of many interacting inflammatory components. Importantly, the identification and validation of therapeutic agents that can effectively prevent and reverse destructive neuroinflammatory processes in human disease have not yet been achieved.

Among the inflammatory mediators that have been identified, a role of the GPR55 receptor has been reported in multiple experimental models (Masquelier et al., 2018; Saliba et al., 2018; Chen et al., 2022). The GPR55 receptor is an orphan GPCR with a wide distribution in the nervous (Marichal-Cancino et al, 2017) and immune systems (Zhou et al., 2016). Further, upregulation of GPR55 and intensification of downstream signaling have been observed in immune cells treated with GPR55 agonists (Wnorowski et al., 2021). In addition, the GPR55 receptor has recently emerged as a potential drug target for the treatment of neurological disease (Foss et al., 2021; Ippolito et al., 2024). Our interest in GPR55 as an inflammatory mediator and drug target was based on two pharmacological characteristics: 1) the recognition that this pro-inflammatory GPCR has a role in the production of peripheral neuropathy (Staton et al., 2008; Okine et al., 2020; Foss et al., 2021); and 2) the identification of both natural and synthesized cannabinoids as interactors with GPR55 (Marichal-Cancino et al., 2017; Bih et al, 2015). Our current therapeutic interest is in the development of the novel cannabinoid KLS-13019 as a GPR55 antagonist that can prevent and reverse chemotherapy-induced peripheral neuropathy (CIPN) (Foss et al., 2021; Ippolito et al., 2024). The proposed mechanism of action for the intervention of neuropathic pain in CIPN was correlated with the prevention and reversal of neuroinflammation in dorsal root ganglion (Brenneman et al., 2022). As KLS-13019 is a novel cannabinoid drug candidate which was derived from cannabidiol, our choice of the GPR55 receptor as a potential target was based on known antagonist interactions with cannabinoid molecules which act through this GPCR (Sharir and Abood (2010).

Our interest in lysophosphatidylinositol originated in this lipid because it has a recognized role in signal transduction mediated through GPR55. Lysophosphatidylinositol arachidonate in particular was reported to be an agonist for GPR55 that resulted in an inflammatory response (Yamashita et al, 2013). In recent studies, we have focused on (LPI-20:4)) as an endogenous pro-inflammatory agent that produced increases in neurons in two primary neuronal culture systems. In these model systems, LPI-20:4 also increased the immunoreactive levels of GPR55 in both dorsal root ganglion and hippocampal cultures (Brenneman et al., 2022). Further, KLS-13019, GPR55 antagonist, has been shown to decrease LPI-20:4-mediated increases in neuritic IL-1b and NLRP3, a component of inflammasome-3.

In the present study, the inflammatory responses of various LPI structures were compared in two GPR55-expressing model systems: dissociated cultures derived from rat embryonic hippocampus and dorsal root ganglion. As LPI exists as a family of structurally-related molecules that very in the length and unsaturation of their fatty acyl component, the fundamental variable examined in our study was the differential effects of these LPI fatty acyl structures on neuroinflammation. While the effects of fatty acyl groups have been recognized to confer major differing biophysical properties for many lipid classes, their role in LPI is now specially examined in a highly defined area of neuroinflammation as measured through a high content fluorescent imaging. With this technique, LPI-mediated effects were restricted to neurons only, with responses in both cell bodies and neurites compared. In the studies to be described, fatty acyl structure is reported to be the major determinant of LPI-mediated inflammatory responses in both hippocampal and dorsal root ganglion cultures. These studies suggest that LPIs should be examined further as inflammatory mediators for their role in neurological disease, with particular importance given to the complexity of LPI fatty acyl structures.

## Materials and Methods

### Materials

All lysophosphatidylinositol (LPI) reagents were obtained from Avanti Polar Lipids (avantilipids.com). LPI-18:1 (85100), LPI-16:0 (85102), LPI-18:0 (85104), LPI-20:4 (85104) LPI-13:0 (850101) and LPI-17:1 (850103) were obtained in sealed ampules and stored at −20 degrees C until used. Stock solutions of all LPI reagents were dissolved in dimethyl sulfoxide to a concentration of 10 mM immediately before use. Serial dilutions then were made with Dulbecco’s phosphate buffered saline (DPBS) to achieve the desired final treatment concentrations. Alamar blue HS Cell Viability Reagent (A50101) was obtained from Invitrogen (Eugene, Oregon). Rat nerve growth factor-b (N2513) was obtained from Sigma Millipore.

### Culture models

Dissociated dorsal root ganglia (DRG) cultures derived from embryonic day 18 rats were employed as the assay system to the measure the inflammatory and anti-inflammatory responses of LPI with various fatty acyl structures. In brief, rat DRG were obtained commercially through TransnetYX (Springfield, IL) and cultures prepared according to methods described previously (Brenneman et al., 2019). Tissue was dissociated with a papain-based kit from Worthington Biochemical Corporation (Lakewood, NJ). The DRG cells were plated at low density (10,000 cells / well) in a 96-well format and maintained in serum-free medium consisting of Neurobasal Medium Plus and rat Nerve Growth Factor-b at 12 ng/ml. Poly-D-lysine coated plates (BD Biosciences, Franklin Lakes, NJ) were employed for this culture system. Prior to the initiation of experiments on day 12 in vitro, a complete change of medium was performed in a working volume of 100 μL.

LPI treatments also were studied in another GPR55 receptor-expressing system (rat hippocampal cultures) that were prepared by methods previously described (Brenneman et al., 2018). Hippocampal tissue was obtained commercially through TransnetYX (Springfield, IL). Tissue was dissociated with a papain-based kit from Worthington Biochemical Corporation (Lakewood, NJ). The hippocampal neurons were platted at low density (10,000 cell/well) in a 96-well format and maintained in serum-free medium consisting of Neurobasal Medium Plus (Thermofisher) with B27 Plus Supplement. Poly-L-lysine coated plates (BD Biosciences, Franklin Lakes, NJ) were used for this preparation. Prior to the initiation of LPI treatment on day 12 in vitro, a complete change of medium was performed in a working volume of 100 μL.

### Immunofluorescent assays

To assess the effects of various LPI treatments, immunofluorescent methods were used to measure neuronal responses in DRG and hippocampal cultures. The goals for these assays included: 1) identification of neuronal structures with antibodies to beta-3 tubulin; 2) to assess the immunoreactive spot area of selected molecular targets (GPR55, NLRP3 and IL-1b) with their respective primary antibodies and distinctively labeled secondary antibodies; 3) to compare the relative responses of the molecular targets in both neuronal cell bodies and neurites. Additional assays that measured immunoreactive areas for IL-6, IL-18 and TNF-a were done to compare the responses of LPI- 20:4 and LPI-18:1. Concentration-effect experiments for LPIs were conducted with a log range from 1 pM to 1 μM for 6 hours.

Prior to fixation, growth medium was removed and the wells were rinsed twice with 100 μL DPBS (37° C). This warm rinse is particularly important to maintain structural stability of neurites. After removal of the DPBS, cultures were fixed for 20 min at room temperature with 50 μL / well of 3.5% formaldehyde (Sigma-Aldrich: 252549) in warm (37°C) DPBS that contained 5.5 μg/mL of Hoechst 33342 dye (Invitrogen: H3570) to label cell nuclei. After removal of the fixative, the cultures were rinsed 3 times with 100 μL of DPBS and then a permeabilization - blocking buffer containing 5% normal goat serum and 0.3% triton-X100 in DPBS was added to the cultures for 10 min. After removal of the blocking buffer, the cultures were rinsed twice with 100 μL of DPBS and then primary antibodies were added for one-hour incubation with shaking at room temperature. Neurons were identified with antiserum to beta-3 tubulin (tuj 1) to measure changes in all neuronal structure parameters. The primary antiserum employed was a rabbit polyclonal obtained from Sigma –Aldrich (T2200) and used at 1:200 dilution. The secondary antibody was an Alexa Fluor 488-conjugated Fab fragment of goat anti-rabbit IgG obtained from Life Technologies (A11070) used at 1:600 for 30 minutes. After the secondary antibody treatment, cultures were rinsed 3 times with 100 μL of DPBS before performing high content fluorescent analysis. For storage prior to image analysis, the wells were placed in 100 μL of sterile DPBS, with the plates wrapped in aluminum foil and maintained at 4° C. For the detection of inflammatory markers, the following primary antibodies were used: IL-1b (PA5–88078); NLRP3 (PA5–79740); and for GPR55 (ab203663). Primary antibodies for IL-1b and NLRP3 were obtained from Life Technologies. The GPR55 antibody was obtained from Abcam. Primary antibodies were diluted 1:250 and all secondary antibodies were used at 1:600. The secondary antibodies were obtained from Life Technologies. The following Alexa Fluor dyes labeled the secondary antibodies: Alexa Fluor 488 (A11070), 555 (A32732), 687(A32733) and 750 nm (A21039). By using secondary antibodies with differing dyes, the same culture wells were assayed for multiple molecular targets. Primary antibodies to IL-18 (10663–1-APP from Protein tech), IL-6 (PA1–26811 from Invitrogen) and TNF-a (bs-2081R) from Bioss) were used at 1:300 dilution. The secondary antibodies for IL-6, IL-18 and TNF-a were Alexa Fluor 555 (A32732), 687(A32733) and 750 nm (A21039), respectively from Thermo Fisher. Secondary antibodies were used at 1:600 dilution.

### High content image analysis

The immunofluorescent assays were conducted on the Cell Insight CX5 high content imaging system (Thermofisher Scientific). This system is based on an inverted microscope that automatically focuses and scans fields of individual culture wells using a motorized stage at predetermined field locations. Fluorescent images from individual fields (895μm × 895μm) were obtained with a 10 x (0.30NA) Olympus objective and Photometrics X1 CCD camera, with analysis by HCS Studio 2.0 Software. The light source was LED with solid state five-color light engine used with filter sets that had the following excitation/emission: 386/440, 485/521, 560/607, 650/694 and 740/809). With this capability, multiple fluorescent assays in a single well were conducted. Images were acquired in a low-resolution mode (4 × 4 binning). Image analyses for neuronal cell bodies and neurites were performed with the Cellomics Neuronal Profiling BioApplication. For analysis of neurons, objects were identified as cells if they had valid nuclei and cell body measures based on size, shape and average intensity. Acceptable ranges were determined in preliminary studies to ensure that aggregated neurons and non-cellular objects were excluded from the analysis.

For both the DRG and hippocampal cultures, the goals were to examine the immunoreactive (IR) spot areas for all the analytes of neurons only. Because the neuronal morphology was different between the two cultures, unique size and shape parameters for cell bodies and neuritic arbors were empirically determined for each culture type in preliminary studies. Once these parameters were determined, the analyses for each culture type were used throughout their respective experiments. Important to these analyses, an essential goal was to compare the immunoreactive spot areas for all analytes in both cell bodies and neurites. Beta-3 tubulin immunoreactivity was used to identify the neurons for each culture type (Brenneman et al., 2022). Ten predetermined fields of view were sampled in each of six replicate wells per plate, with two replicate plates used for each assay. The age of the DRG cultures at the time of analysis was 12 days after plating. Because primary cultures exhibit a variety of neuronal phenotypes and a range of morphological complexities, extensive sampling was employed to obtain an average neuronal response with the inflammatory markers. For measuring parameters of beta-3 tubulin immunoreactivity and spot analysis for the inflammatory markers, the Cellomics Neuronal Profiling Bioapplication was used that combined spot area analysis on neurons that resided within this BioApplication. For analysis of spot immunoreactivity with this BioApplication, a key parameter was the empirical establishment of fluorescent thresholding that permitted the use of a dynamic range that optimized the measurement of fluorescent differences among the treatment groups as well as distinguishing the fluorescent signal from background. This thresholding level was set based on our previous experience with antibody-based assays and the smallest distinguishable size of immunoreactive spots (radius:1.5 μ) under our imaging conditions. With this algorithm, the immunoreactive area was a relative measure that was characterized by an effective computerized spot analysis in a rapid screening mode. Importantly, the same imaging parameters for neurons from all treatment groups were employed. The key comparisons in these studies were aimed at measuring the changes in immunoreactive area that were associated with LPI treatments. Due to the observed differences in the cellular distribution among the analytes, the cellular locations of all inflammatory markers were determined, thus distinguishing the relative changes between cell bodies and neurites. This capability and experimental focus were obligatory aspects of measuring the inflammatory markers by image analysis. Again, the goal of the studies was to obtain quantitative measures of the relative changes in immunoreactive area for each of the analytes after LPI treatment. An estimate of 1000–1300 neurons were assessed for each treatment. Because our goal was to compare and present multiple assays among treatments, the data were expressed as the percent of control. In all cases, the results were measured as immunoreactive area (μ^2^) for each of the analytes per neuron. The presentation of the data was expressed as a percentage of their respective controls to permit a comparative analysis of among the various immunofluorescent assays. To provide further detail for these high content analyses, the control values obtained for each assay in both DRG and hippocampal cultures are presented in Table 1 for comparative purposes.

### Viability assay

Before the fixation of cultures to be assessed by image analysis for inflammatory markers, a viability assay using alamar blue was conducted. For this assay, 10 μl of the dye was added directly to the culture well that contained 100 μl of nutrient medium. Incubation time with the dye was 2 hours and absorbance measured at 570 nm. After removal of the dye, the cultures rinsed with 100 μl of DPBS before subsequent fixation and follow-up immunofluorescent assays. On every plate, wells without cells were used to provide a blank reading that was used to subtract background absorbance.

### Statistical Analysis

All statistical comparisons involving 3 or more groups were made by ANOVA, with normality of values tested with the Shapiro-Wilk test followed by a multiple comparison of means test with the Holm-Sidak method as performed through Sigma Plot 16. Statistical comparisons between two groups were tested with a two-tailed t-test.

## Results

### LPI Structure Determines Neuroinflammatory Responses

**Figure 1:**
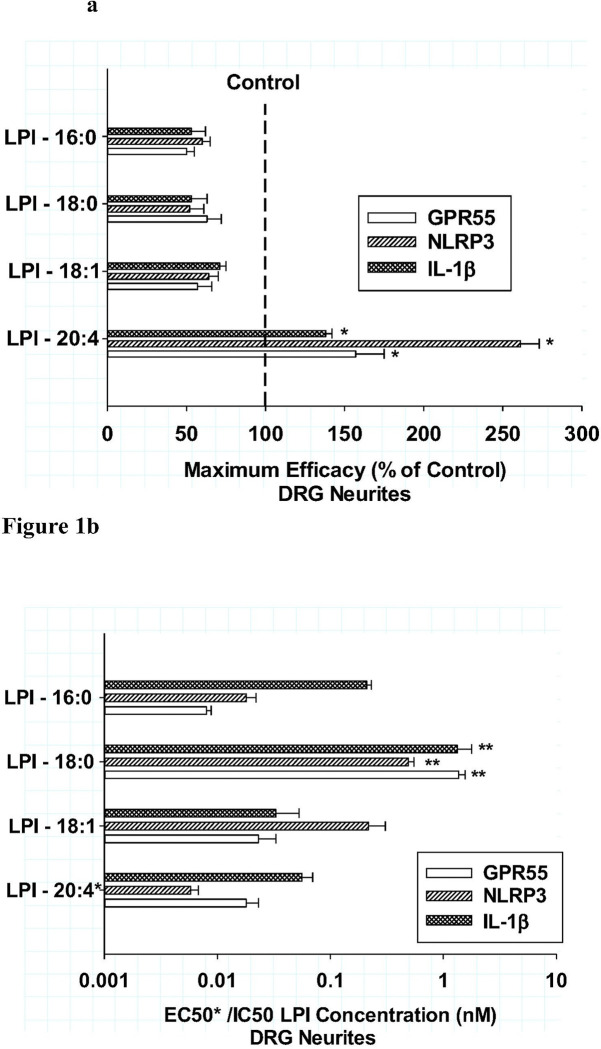
**ab** Effects of various lysophosphatidylinositol fatty acyl structures on *neuritic* area for GPR55 (open bar), NLRP3 (hatched bar) and IL-1b (cross-hatched bar)in cultured DRG neurons after treatment for 6 hours **Fig.1a**: The *maximum efficacies* of four LPI structures were compared as a percentage of their respective controls. Each value is the mean of 12 well determinations from two separate experiments and each well was assessed from 10 microscopic fields. The error bar is the standard error. The dotted vertical line depicts the control. The maximal efficacies of LPI-16:0, LPI-18:0 and LPI-18:0 were all significantly different from LPI-20:4 *(P < 0.001), but not different from each other for all the inflammatory markers. **Fig.1b:** The *potencies* of LPI structure were compared as either their *EC50 for LPI-20:4 or their IC50s for all other LPIs for each of the analytes. Only LPI-18:0 was significantly less potent in comparison to all the other LPIs for all three inflammatory markers **(P<0.004).

To explore differential effects of LPI-fatty acyl structures on neuroinflammatory responses, high content fluorescent imaging analyses of *neuritic* GPR55, NLRP3 and IL-1b levels in DRG cultures were measured initially after a 6-hour incubation with the following LPI fatty acyl compositions: 16:0, 18:0, 18:1 and 20:4. As shown in Fig. 1a, the maximum efficacy of all four LPI structures are compared as a percentage of their respective controls. Confirming previous observations (Brenneman et al, 2022), LPI- 20:4 significantly increased the levels of all three inflammatory measures (P < 0.001) in comparison to their controls, with NLRP3 exhibiting the greatest (2.6-fold) increase from control. For GPR55 and IL1b, there was a mean increase of 1.5-fold from control. In contrast, significant decreases (P < 0.001) in efficacy were observed with LPI structures comprised of 16:0, 18:0 and 18:1. The average decrease (58 ± 2% of control) in efficacy for neuritic levels of GPR55, NLRP3 and IL-1b were not significantly among the three LPI structures. The potencies of the various LPI structures were also measured for the three neuroinflammatory measures as shown in Fig. 1b. EC/IC50 potencies were calculated with a 4-parameter logistic analysis of the responses. All four LPI structures exhibited potent (10 pM to 1nM) responses, with LPI-18:0 being the least potent of the four structures (P < 0.001), while the others responded similarly. These data indicated that despite the opposite pharmacological response of LPI-20:4 in comparison to the other LPI structures, the potencies of LPI-16:0 and LPI-18:1 were not statistically different among the three neuroinflammatory markers, with the exception of LPI-18:0 which was significantly less potent P < 0.01).

**Figure 2:**
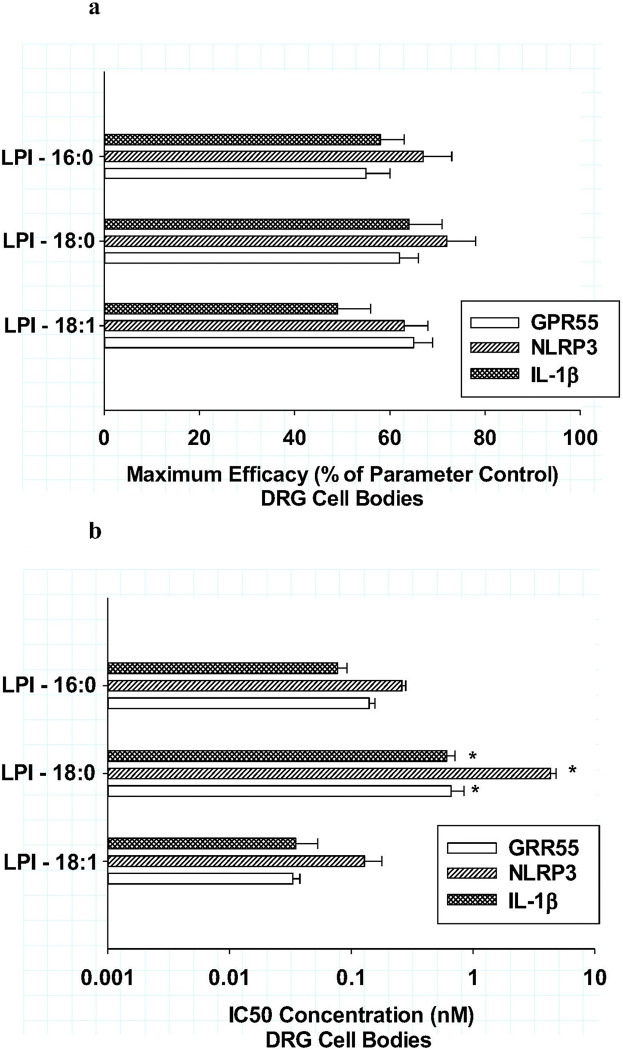
**ab** Effects of various lysophosphatidylinositol fatty acyl structures on *cell body* areas for GPR55 (open bar), NLRP3 (hatched bar) and IL-1b (cross-hatched bar)in cultured DRG neurons after treatment for 6 hours **Fig.2a:** The *maximum efficacies* of the three anti-inflammatory LPI structures were compared as a percentage of their respective controls. Each value is the mean of 12 well determinations from two separate experiments and each well was assessed from 10 microscopic fields. The error bar is the standard error. No significant differences were observed among these three LPIs for any of the inflammatory markers. **Fig 2b:** The potencies of the three anti-inflammatory LPI structures were compared for each of the analytes. Only LPI-18:0 was significantly less potent in comparison to all the other LPIs for all three inflammatory markers *(P<0.003).

**Figure 3:**
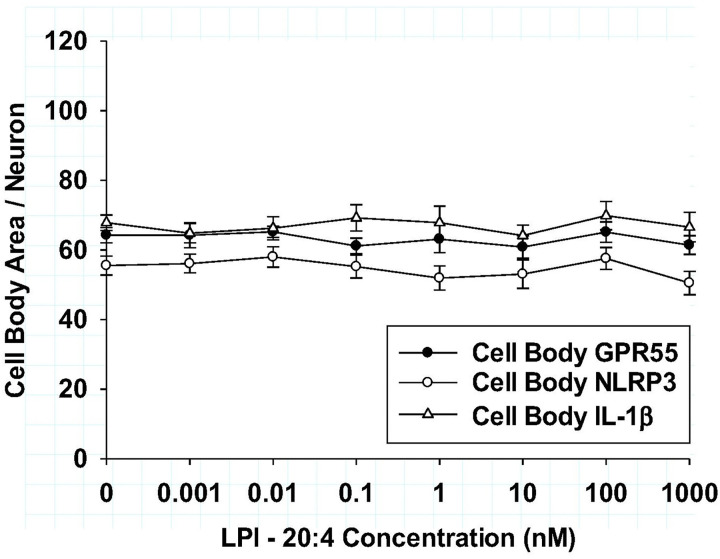
Effects of lysophosphatidylinositol-20:4 on *cell body* areas for GPR55 (closed circle), NLRP3 (open circle) and IL-1b (open triangle) in cultured DRG neurons after treatment for 6 hours In contrast to the anti-inflammatory effects of various LPIs in figure 2ab, there were no significant effects produced by LPI-20:4 for the three analytes in cell bodies. Each value is the mean of 12 well determinations from two separate experiments and each well was assessed from 10 microscopic fields. The error bar is the standard error.

As shown in Fig. 2a, a similar analysis of the three anti-inflammatory LPI structures as determined from the neuritic analysis above were also measured in *cell bodies* for GPR55, NLRP3 and IL-1b levels in DRG cultures. Like the neuritic analysis, significant decreases in all three inflammatory markers were observed in cell bodies after treatment with LPI-16:0, LPI-18:0 and LPI-18:1. The mean efficacy of these decreases for the three anti-inflammatory LPI structures was 61 ± 2%, which was not significantly different from that observed for the neuritic analysis. The potencies of the three anti-inflammatory LPI structures are shown in Fig. 2b, with LPI-16:0 and LPI-18:0 exhibiting high potencies (30–300 pM) and LPI-18:0 again exhibiting the least potent (1–3 nM) responses in the DRG cultures. As shown in Fig. 3, a similar analysis with the pro-inflammatory LPI-20:4 indicated no significant changes in cell body inflammatory markers over a wide range of concentration from 1pM to 1μM. Together, these data indicate that the pro-inflammatory responses of LPI-20:4 were restricted to neurites as determined in DRG neurons; whereas the anti-inflammatory responses to the other LPI structures exhibit their effects on all three inflammatory markers for both neurites and cell bodies.

### Comparison of LPI-mediated neuroinflammation in DRG and hippocampal cultures

**Figure 4:**
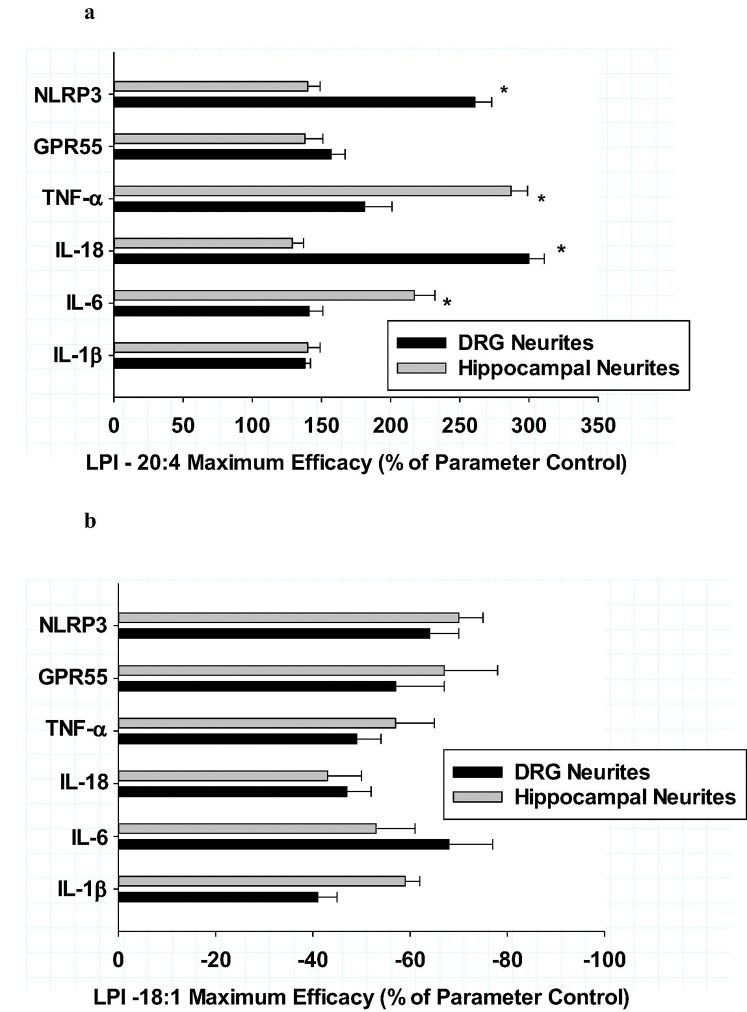
**ab**: Comparison of dorsal root ganglion (dark bars) and hippocampal cultures (grey bars) for their maximum efficacy responses of various cytokine neuritic areas to either LPI −20:4 (**Fig 4a**) or LPI-18:1 (**Fig. 4b**) after 6 hours of treatment Significant differences *(P<0.001) between the two cultures types were observed for NLRP3, TNF-a, IL-18 and IL-6 after treatment with LPI-20:4 as shown in Fig. 4a. Therse were no significant differences in maximum efficacies between the two culture types after treatment with LPI-18:1 as shown in Fig.4b.

Because the previous section demonstrated that various LPI structures were shown to have distinct pro-inflammatory or anti-inflammatory responses, additional studies were conducted with an expanded comparison of the archetypal, pro-inflammatory LPI-20:4 with the anti-inflammatory LPI-18:1, which had the greatest potency in reducing both neuritic and cell body IL-1b in DRG cultures. For these further analyses with additional cytokine responses, a comparison was made between two primary neuronal culture systems that naturally express the LPI receptor GPR55: hippocampal and DRG cultures. As shown in Fig. 4a, the maximum efficacy of LPI-20:4-mediated increases in various inflammatory mediators are demonstrated for DRG cultures (dark bars) and hippocampal cultures (gray bars). These data indicated that while both culture types exhibited increases after LPI-20:4 treatment, the magnitude of the increases differed between the two culture types for some of the following inflammatory mediators: NLRP3, TNF-a, IL-18 and IL-6. Enhanced increases from controls were observed in DRG cultures for NLRP3, and IL-18. In contrast, enhanced increases from controls were observed in hippocampal cultures for TNF-a and IL-6. However, there were no differences in the increases from controls between the two culture types for the GPR55 receptor and IL-1b. These data indicated that the responses to LPI-20:4 were not uniform between DRG and hippocampal cultures; rather the pro-inflammatory agent had maximum efficacy responses that were different and reproducible.

In contrast to the effects of the LPI-20:4 induced differences in maximum efficacies for various inflammatory agents, experiments comparing the maximum *anti-inflammatory efficacy* responses after treatment with LPI-18:1 showed no significant differences among the various inflammatory mediators as shown in Fig. 4b. The mean anti-inflammatory responses for LPI-18:1 was a decrease of 56 ± 4% of control for all inflammatory markers. Further, the mean anti-inflammatory responses did not differ between DRG (54 ± 4) and hippocampal cultures (58 ± 4) % of control. Taken together, while there were significantly different responses between DRG and hippocampal cultures in regard to LPI-20:4-mediated increases in the inflammatory mediators, there were no significant changes in efficacy in response to the anti-inflammatory responses to LPI-18:1.

**Figure 5:**
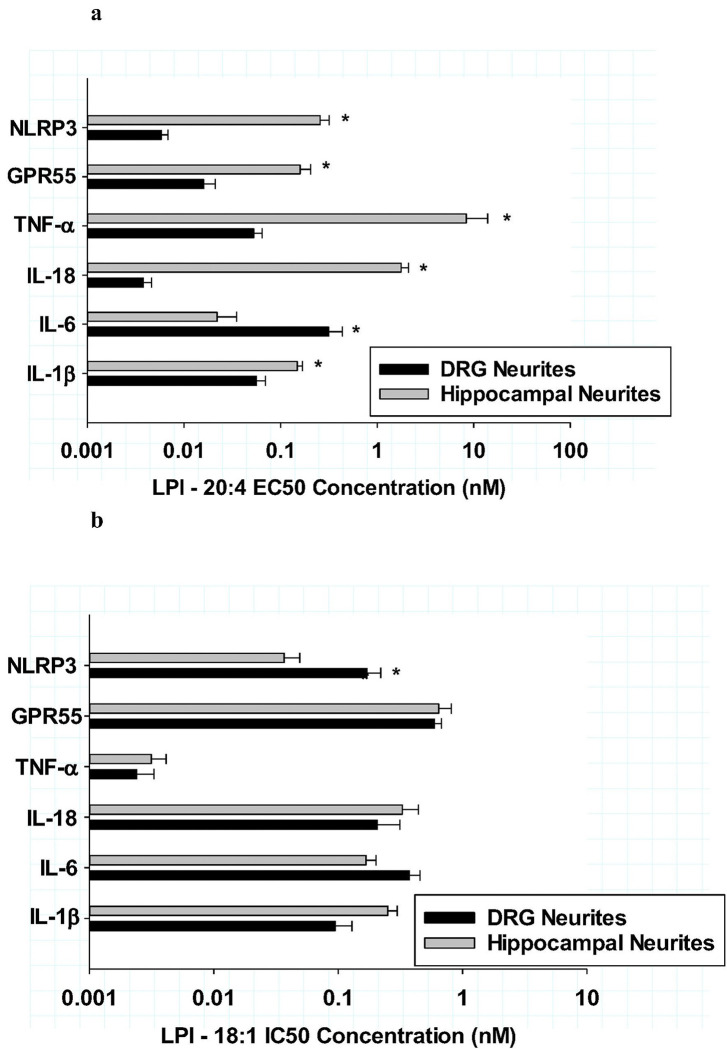
**ab:** Comparison of dorsal root ganglion (dark bars) and hippocampal cultures (grey bars) for their potency of responses from various cytokine neuritic areas after treatment with either LPI −20:4 (**Fig 5a**) or LPI-18:1 (**Fig. 5b**) after 6 hours of treatment In Fig5a, significant differences *(P<0.001) between the two cultures types were observed for measures except IL-1b after treatment with LPI-20:4. Each value is the mean of 12 well determinations from two separate experiments and each well was assessed from 10 microscopic fields. The error bar is the standard error. In Fig5b, the only significant difference between the two cultures types after treatment with LPI-18:1 was with NLRP3 * (P<0.01). In comparison of responses among the 6 cytokines, only TNF-**a** exhibited a significant (p<0.001) increase in potency after treatment with LPI-18:1 in both culture types.

Similar comparisons between culture types were conducted in regard to the potencies of the responses to LPI-20:4 and LPI-18:1 for DRG cultures (dark bars) and hippocampal cultures (grey bars) as shown in Figs. 5a and 5b, respectively. High potency was observed after LPI-20:4 treatment for all the inflammatory markers in DRG cultures, with EC50s ranging from 3–300 pM. While the LPI-20:4-mediated increases in all the inflammatory markers were also potent (22 pM to 8 nM) in hippocampal cultures, the EC50s were all significantly less potent than that observed in DRG cultures, with the exception of IL-6. LPI-18:1 potencies for various anti-inflammatory responses between DRG and hippocampus were compared as shown in Fig. 5b. These comparisons indicated that the mean IC50s for LPI-18:1 for all six markers were 0.240 + .09 nM for DRG cultures and 0.238 + .09 nM for hippocampal cultures. Among the six inflammatory makers in both culture types, only TNF-a exhibited an IC50 for LPI-18:1 that was exceptionally potent at 3 pM.

Because the responses to LPIs were often expressed as a percentage of the individual parameter control for various inflammatory markers to allow for a comparison of relative effects of multiple analytes, the values for control levels for each marker with both culture types are presented in Table 1. These values compare non-LPI stimulated levels of inflammatory markers to give added detail to these two LPI-responsive culture systems. With the exception of TNF-a, DRG cultures exhibited lower levels of the inflammatory markers in cell bodies in comparison to that in hippocampal cultures (P < 0.002). However, a comparison of all inflammatory markers in neurites, there was no statistically significant difference (P < 0.06) observed between DRG cultures as compared to that in hippocampal cultures. If the responses to individual inflammatory markers areas were compared for neurites between the two culture types, significant (P < 0.02) increases in GPR55, NLRP3 and IL-18 and were observed in hippocampal cultures as compared to the DRG cultures. In contrast, significant (P < 0.001) increases in IL-6 and TNF-a, were observed in control DRG cultures as compared to hippocampal cultures. No significant differences in the neuritic area/neuron for controls were observed only for IL-1b between the two cultures types. To present and compare the area ratios for various immunoreactive areas, these data are presented also in Table 1. Thus, the control levels of inflammatory neuritic areas were dependent on the individual markers between the two culture types.

**Figure 6:**
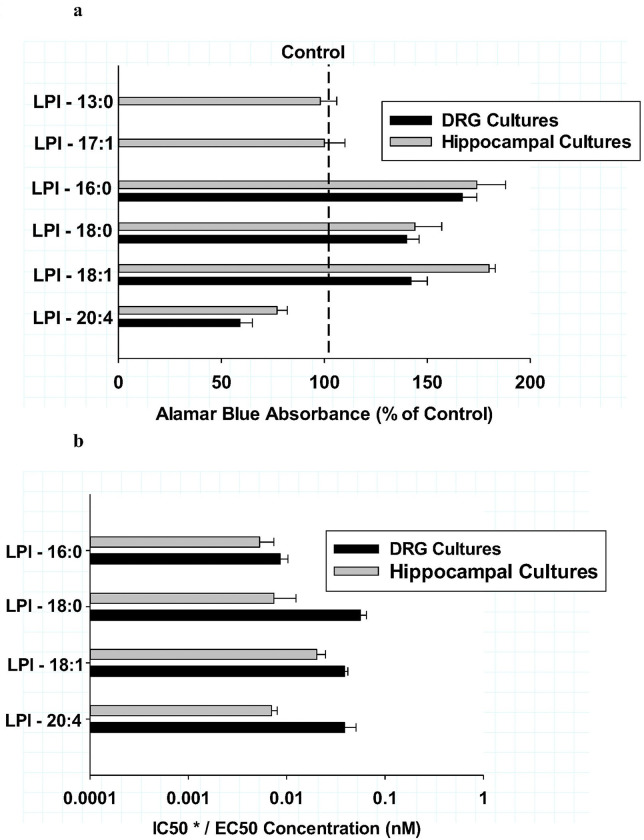
**ab:** Effects of various lysophosphatidylinositol fatty acyl structures on the alamar blue viability assay after treatment for 6 hours: comparison of dorsal root ganglion (dark bars) and hippocampal cultures (grey bars) **Fig.6a**: The maximum efficacy of LPI structures were compared as a percentage of their respective controls. Each value is the mean of 12 well determinations from two separate experiments. The error bar is the standard error. The dotted vertical line depicts the control. The maximal efficacies of LPI-16:0, LPI-18:0 and LPI-18:0 were all significantly greater than that observed from LPI-20:4, LPI −13:0 and LPI −17:1*(P < 0.001) for hippocampal cultures. The mean values of LPI −13:0 and LPI – 17:1 were significantly greater that that observed after treatment with LPI – 20:4 **(P< 0001). Statistical comparisons of individual LPIs and cultures: DRG: LPI-20:4 < LPI −18:1 = LPI −18:0 < LPI-16:0 (P< 0.02) Hippocampus: LPI – 20:4 < LPI - 18:0 < LPI −18:1 = LPI −16:0 (P< 0.05). **Fig.6b**: The potencies of LPI structure were compared as either their *IC50 for LPI-20:4 or their EC50s for all other LPIs for the alamar blue viability assay Statistical comparison of individual LPIs and cultures were: DRG: LPI-16:0 < LPI-18:0 = LPI-18:1 = LPI-20:4 (P<0.05). Hippocampus: LPI-18:1 = LPI-20:4 = LPI-18:0 = LPI-16:0. (P<0.05).

In order to obtain an additional measure of cellular viability responses of primary neuronal cultures after various LPI treatments, the alamar blue assay was employed. This assay was conducted prior to the analyses of fixed cultures focused on antibody-based inflammatory markers. As shown in Fig. 6a, the four LPI structures were compared for the effects on the alamar blue assay of the LPI-mediated effects on maximal efficacy as determined from a concentration range from 1 pM to 1 μM. From these viability studies, only LPI-20:4 was shown to significantly decrease (P < 0.001) the maximal efficacy observed for both DRG (dark bars) and hippocampal cultures (gray bars) as a percentage of their respective controls. The combined mean decreases in the alamar blue assay for both culture types was 68 ± 6% of control.

In contrast, LPI structures with fatty acyl structures of 16:0, 18:0 or 18:1 produced significant increases (P < 0.001) in the alamar blue assay in comparison to their respective controls as shown in Fig. 6a. The mean increase for these three LPI structures was 150 ± 20 (% of control). Further, there were no significant differences in the maximal efficacy of the three LPI structures that exhibited the increases. The potency of the four LPI structures in the alamar blue assays were compared as shown in Fig. 6b. In the case of LPI with fatty acyl groups 16:0. 18:0 or 18:1, the EC50 for increases from control was shown to be similar with a mean response of 10 pM for hippocampal cultures and 30 pM for DRG cultures. The mean EC50 response between the two cultures types was not significantly different. In contrast, LPI-20:4 treatment in the alamar blue assay resulted in a decrease that was significantly different from control (P < 0.001). The IC50 of this decrease produced by LPI-20:4 was not different from the mean EC50 observed with LPI-16:0, LPI-18:0 and LPI-18:1.

While all of the endogenous LPI structures produced potent and robust changes in the alamar blue assay, additional control LPI agents that had non-endogenous fatty acyl components were also tested in the alamar blue assay in hippocampal cultures. LPI – 17:1, a plant-derived lipid, was employed to test if a non-mammalian fatty acyl group would produce any effect with the alamar blue assay. In addition, LPI – 13:0, a gut-derived fatty acyl group, was also tested. As shown in Fig. 6a, treatment with either LPI-13:0 or LPI-17:1 produced no significant differences from control culture values.

**Figure 7:**
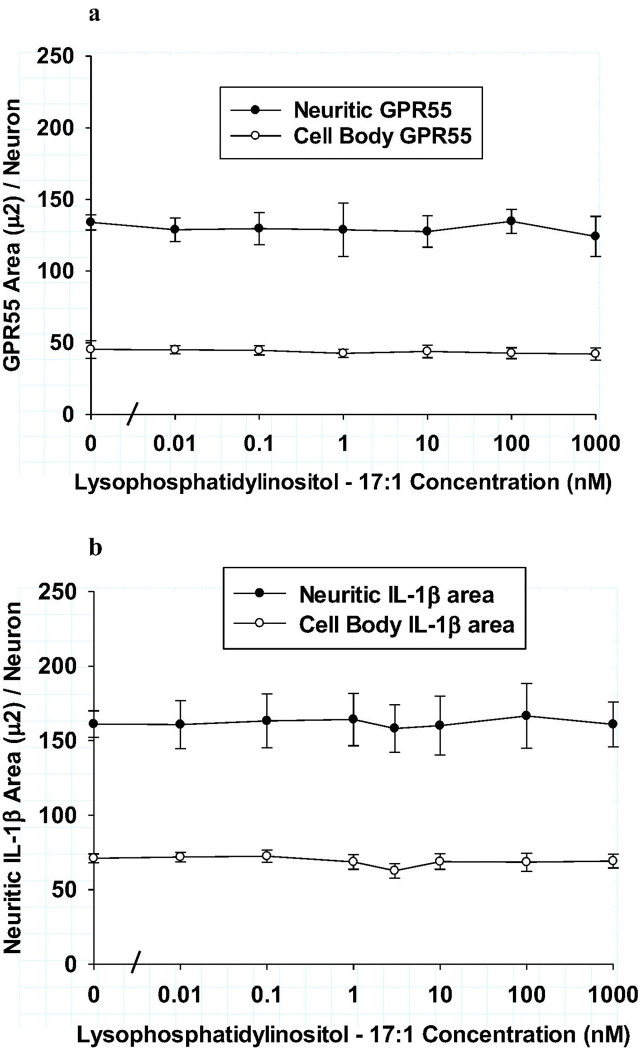
**ab:** Comparison of lysophosphatidylinositol-17:1 on neurites (closed circles) and cell bodies (open circles) of hippocampal cultures after 6 hours treatment **Fig. 7a:** Effect of LPI-17:1 on GPR55 immunoreactive area per neuron. Each value is the mean of 12 well determinations from two separate experiments and each well was assessed from 10 microscopic fields. The error bar is the standard error. No significant changes from that of control were observed for either neurites or cell bodies. **Fig. 7b:** Effect of LPI-17:1 on IL-1b immunoreactive area per neuron. Each value is the mean of 12 well determinations from two separate experiments and each well was assessed from 10 microscopic fields. The error bar is the standard error. No significant changes from that of control were observed for either neurites or cell bodies.

**Figure 8:**
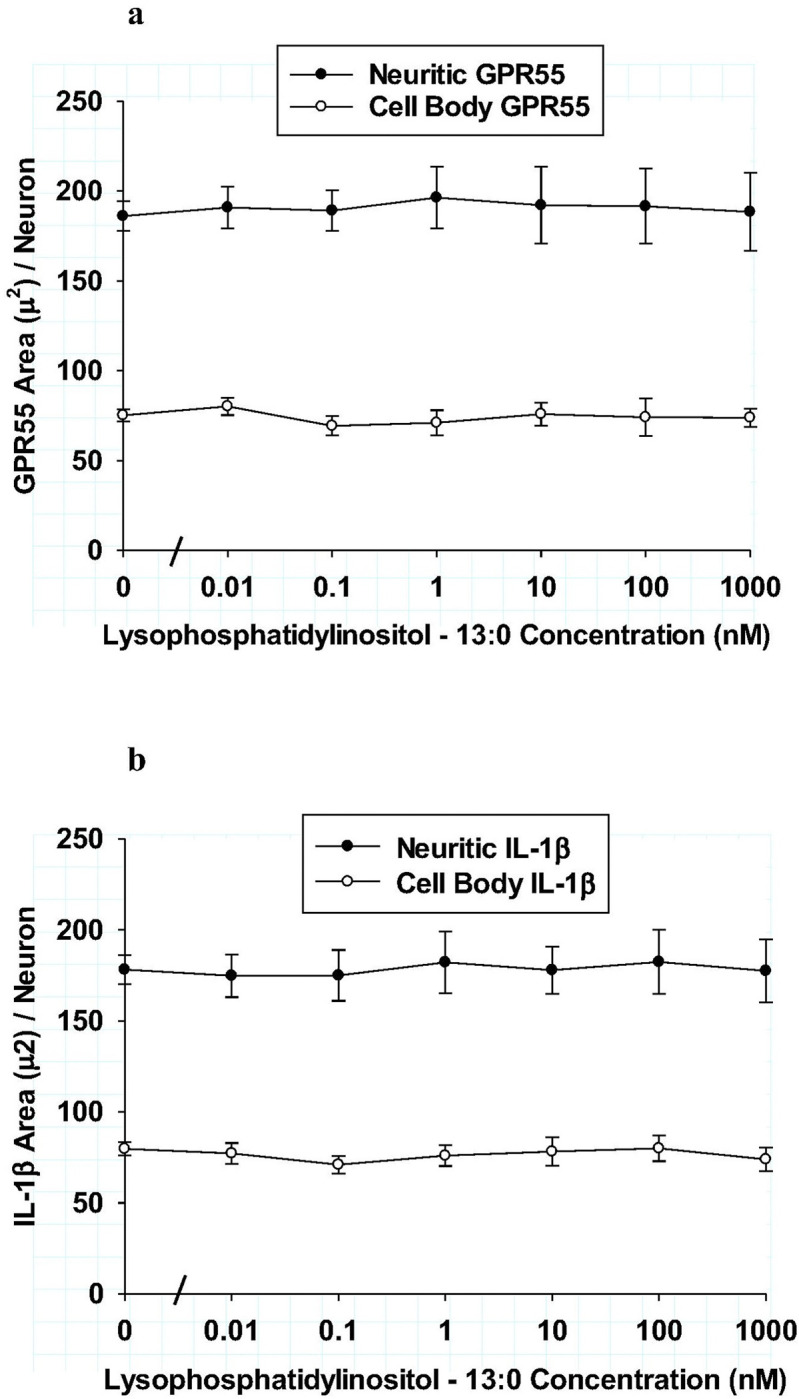
**ab:** Comparison of lysophosphatidylinositol-13:0 on neurites (closed circles) and cell bodies (open circles) of hippocampal cultures after 6 hours treatment. **Fig. 8a:** Effect of LPI-13:0 on GPR55 immunoreactive area per neuron. Each value is the mean of 12 well determinations from two separate experiments and each well was assessed from 10 microscopic fields. The error bar is the standard error. No significant changes from that of control were observed for either neurites or cell bodies. **Fig. 8b:** Effect of LPI-13:0 on IL-1b immunoreactive area per neuron. Each value is the mean of 12 well determinations from two separate experiments and each well was assessed from 10 microscopic fields. The error bar is the standard error. No significant changes from that of control were observed for either neurites or cell bodies.

The control LPI agents that had non-endogenous fatty acyl components were also tested for possible effects on neuroinflammatory markers in hippocampal cultures. As shown in Fig. 7a, LPI – 17:1 was tested for effects on GPR55 immunoreactive area in both neurites and cell bodies in hippocampal cultures. Using the same concentration range and assay conditions as employed with all other LPIs, no significant differences from control were observed for either neurite GPR55 area or that in cell bodies. As shown in Fig. 7b, the effect of LPI – 17:1 on IL-1b immunoreactive area was tested in neurites and cell bodies. Again, LPI – 17:1 had no significant effect on IL-1b responses after 6 hours of incubation. Because of the importance in demonstrating the specificity of endogenous LPI fatty acyl structures, an additional non-endogenous LPI was tested with a 13:0 component. As shown in Figs. 8a and 8b, LPI – 13:0 treatment produced no significant changes in either the GPR55 receptor or IL-1b in hippocampal cultures. Together, these studies indicated while endogenous LPI structures were found to produce either a pro-inflammatory or an anti-inflammatory effect in neurons, treatment with two control LPI structures produced no effect on GPR55 or IL-1b. Thus, fatty acyl structure was concluded to be the essential determinant feature of LPI that mediates the neuroinflammatory responses tested.

### Immunofluorescent imaging of cytokines

Although the emphasis of the LPI studies was to measure the average high content changes of immunoreactive area for various neuroinflammatory substances resulting from over 1000 neurons per concentration-treatment, this technique also permits the single field display of immunofluorescent images based on the various cytokines to assess cellular labeling patterns and potential overlap of various analytes after LPI treatment. While previous reports from our group had shown morphological immunofluorescent (IF) images for GPR55, NLRP3 and IL-1b in DRG cultures, the current studies were confined to TNF-a, IL-18 and IL-6 on DRG neurons.

**Figure 9:**
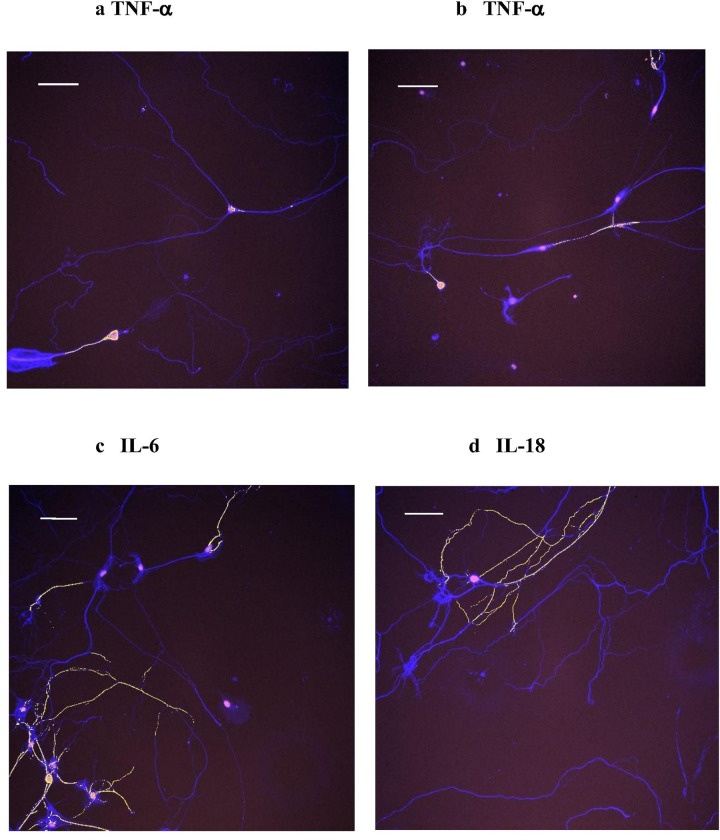
Immunofluorescent images of cytokines in DRG cultures from high content analysis. The calibration bar is 100 m for all panels. Immunoreactive cytokine spots are shown in yellow, beta tubulin-4 IR is shown in purple and the nuclei are shown in pink for all panels. All images were obtained from control DRG cultures on day 11. **Figs.9ab**: TNF-aimmunofluorescent neurons are shown. **Fig9c**: IL-6 immunofluorescent neurons are shown. **Fig.9d**:IL-18 immunofluorescent neurons are shown.

Because the high content analysis shown in Figs. 5a and 5b indicated that there were apparent differences in the potency of both LPI-20:4- and LPI-18:1-mediated effects on neuritic TNF-a in comparison to other inflammatory markers, further morphological studies were conducted with this cytokine on DRG cultures. These high potency effects on TNF-a responses were confined to DRG for LPI-20:4 and to both culture types for LPI-18:1. As shown in Fig. 9a, the TNF-a IF in this field showed an intense labeling of TNF in an initial segment of a proximal neuronal process and an annular ring of labeling around the perimeter of the cell body. As the morphological effects on neurites were variable among individual neurons, a report on these LPI-mediated changes will be the subject of future work. In Fig. 9b, another TNF-a IF image is shown with the same labeling pattern in the cell body and the proximal process segment. This two-feature labeling pattern of the cell body and an initial segment was commonly observed with TNF-a. In Fig. 9c, a DRG neuron stained with antibodies to IL-6 showing an extensive labeling of the neuritic arbor as well as annular labeling around the cell body. In Fig. 9d, a DRG neuron stained with antibodies to IL-18 is shown with extensive labeling of the neuritic arbor without annular labeling of the cell body. In general, IL-6 and IL-18 were always observed together on the same DRG neuron, but without a 100% overlapping labeling pattern.

## Discussion

### LPI: a multifactorial regulator of neuroinflammation

The GPR55 receptor has been recognized to be have effects on inflammation in multiple systems (Apweilaret al., 2024; Chen et al., 2022; Minamihata et al., 2020). It is now generally accepted that LPI is an endogenous agonist for GPR55 that has a broad spectrum of biological actions, including inflammation (Oka et al., 2009; Yamashita et al., 2013). In the present studies, we have confirmed that LPI-20:4 increases neuroinflammation in two primary neuronal preparations. However, we now have demonstrated that this action of LPI on neuroinflammation was totally dependent on the fatty acyl composition of this endogenous family of compounds which can mediate either pro-inflammatory or anti-inflammatory actions. Molecular diversity has been reported to occur in mouse brain LPI with the following fatty acyl groups being observed: 20:4, 18:0, 18:1, 16:0 as determined from mass spectrometry studies (Kondo et al., 2022). Similar analysis of rat hippocampus has shown that the major fatty acyl compositions of LPI were 16:0, 18:0 and 20:4 (Miranda et al., 2019). Both the ventral and dorsal hippocampus exhibited similar LPI fatty acyl compositions. In the present study, each of these LPI structures have been examined for their effects on neuroinflammatory responses in two GPR55-expresssing culture systems. To be specific, LPI – 18:1, LPI-16:0 and LPI-18:0 now have been shown to produce high potency, high efficacy anti-inflammatory actions on multiple inflammatory markers including GPR55, NLRP3, IL-6, IL-1b and TNF-a) on neurites from both DRG and hippocampal neurons grown in culture. Significantly, if non-endogenous fatty acyl chains (13:0 and 17:1) of LPI were treated with the neuronal systems, there were no detectible effects on pro-inflammatory or anti-inflammatory responses over a wide range (10 pM to 1μM) of concentrations. Together, these observations indicate that the fatty acyl component on LPI is the sole determinant of this pharmacological action, with only the LPI combined with arachidonic acid being capable of producing a pro-inflammatory response.

With the emergence of LPI-20:4 as the lone structure of LPI that produced an inflammatory response, it was of interest that the regulation of this particular LPI species has been associated with a specific enzyme: lysophosphatidylinositol acyltransferase 1 (LPIAT1). This enzyme preferentially transfers 20:4, with only weak enzymatic activity for transferring saturated and unsaturated acids (Caddeo et al., 2021). Further, LPIAT1 is localized to the mitochondria-associated membrane (Hirata et all, 2024). While our interest has been focused on the potential interruption of homeostasis that may be produced by LPI-20:4, it is evident from knockout studies of LPIAT1 that this specific LPI is necessary for normal neurodevelopment and life span. LPIAT1 −/− mice showed atrophy of cerebral cortex and hippocampus as well as premature death within a month (Lee et al., 2012). Thus, decreasing the expression of LPIAT1 may have more risk to vital physiological processes than decreasing excess LPI-20:4 levels by a pharmacological approach with a cannabinoid-mediated antagonist action at the GPR55 receptor.

### Neuroinflammatory cytokine responses

In the present study, our goals were both to test the roles of various LPI-related structures and to extend the scope of the responding neuroinflammatory cytokines that were linked to LPI treatments. In the latter context, both inflammsome-3 derived cytokines (IL-1b and IL-18) and transcriptionally-regulatory inflammatory cytokines (IL-6 and TNF-a) were examined after treatment with various LPI structures on primary neuronal cells grown in cell culture. The choice of these specific inflammatory cytokines was also based on their known linkage to high levels of reactive oxygen species (ROS) that are generated in mitochondria (Naik and Dixit, 2011). As shown in our previous work, we found that the GPR55 receptor, the molecular target of LPI, could be co-localized with a mitochondrial marker COX4 (Brenneman et al., 2019). While 4 of the cytokines showed a similar expression in control cultures, there were apparent subtle differences in their immunoreactive spot expression among the cytokines. The current high content studies indicated that all of the LPI-mediated changes were apparent in neurites rather than cell bodies. As previously hypothesized, this selectivity for neurites was proposed to be due to the predominance of synaptic mitochondria in the neuritic compartment in comparison to cell bodies (Brenneman et al., 2019; Brenneman et al., 2024). In the present studies, the morphological spot distributions of IL-6 and IL-18 IR were shown to be similar to that observed previously for IL-1b, GPR55 and NLRP3, with expansive cytokine labeling for much of the neuritic arbor (Brenneman et al., 2022). The notable exception to this labeling pattern was observed with TNF-a, in which the immunoreactive spot labeling pattern was predominantly detected in the proximal neuritic process next to the cell body and in an annular labeling pattern present around the cell bodies. Our working interpretation of this spot distribution is that TNF-a is highly localized to the axon initial segment, a specialized area that is known to contain highly dense scaffolding and regulatory proteins that are involved in action potential generation. Such a unique localization of TNF-a may contribute to its known effect on enhanced excitability in sensory neurons of the DRG (Valdez-Morales et al., 2022). Such a pattern would predict that some LPIs may enhance or regulate DRG excitability (North et al., 2019) or neuronal plasticity that occurs with pain (Woolf and Salter, 2000; Jang and Garraway, 2024). It is of interest that LPI has been shown to enhance hippocampal CA1 LTP through the GPR55 receptor (Hurst et al., 2017).

### Agonist versus inverse agonist model

The major finding of the present studies was that the fatty acyl composition of various LPI structures can have opposite actions on neuroinflammation. While the pharmacological explanation of this dualistic outcome has not been established yet, there is evidence in the literature that GPR55 is capable of an inverse agonist action (Badolato et al., 2019; Apweiler et al., 2021). In general, the concept of inverse agonism is based on the classical two-state model for GPCRs that are considered to exist in an equilibrium between active and inactive confirmations (Sato et al., 2016). With this model, inverse agonists shift the equilibrium to the inactive state and thereby suppress basal activity. In the case of GPR55, a model has been reported to identity critical residues that are required for activation of this pro-inflammatory GPCR (Lingerfelt et al, 2017). An important element of this model is the property that the GPCR would be constitutively active to increase neuroinflammatory products, as observed with LPI-20:4. With this model, an inverse agonist would decrease neuroinflammatory actions by shifting the receptor to its inactive state thereby resulting in the observed decreases in cytokine expression in the case of LPI-18:0, LPI-18:1 and LPI 16:0. This was the observed effect for these LPI structures. We would speculate that LPI diversity is a potential mechanism of neuroinflammation which has a role in mediating homeostatic regulation of neuritic cytokine balance in synaptic mitochondria (Brenneman et al, 2024). Further, that lack of a response of LPI treatment on cell body cytokines may again be related to a reduced level of functioning synaptic mitochondria in this cellular compartment, as previously proposed (Brenneman et al., 2024).

To be clear, the diversity of actions associated with various fatty acyl LPI structures has only been examined for neuroinflammation in two model systems. Because of the diversity of GPR55 receptor distribution, the actions described herein may or may not be pertinent to other known actions mediated by this GPCR. However, these data provide compulsive evidence that only a single species of LPI of known fatty acyl composition should be used in the future explorations of GPR55 actions, rather than undefined mixtures of LPI structures from plant or animal sources.

### Alternative lipid-sensing GPCR model

An alternative explanation for the diversity of LPI responses may reside in the possible involvement of other receptors besides GPR55 which could mediate anti-inflammatory outcomes. For example, two other lipid-sensing (L-S) GPCR receptors have been associated with cannabinoids and both have reported effects on anti-inflammatory actions: GPR18 and GPR119 (Irving et al., 2017). These alternative GPCRs now have been briefly evaluated by three criteria relevant to the current studies: 1) the known tissue distribution of these (L-S) receptors; 2) the current evidence that these receptors produce an anti-inflammatory action; and 3) reported responses of these receptors to LPI treatment. In regard to tissue expression, GPR18 has been reported be present in DRG, but not in hippocampus (Honkisz-Orzechowksa et al., 2024). Similarly, GPR119 has been detected in the DRG but not in hippocampus (Zuniga-Romero et al., 2022). Thus, in comparison to the demonstrated high abundance presence for the GPR55 receptor in both DRG and hippocampus, the cited localization criterium would not yet support the alternate association for these receptors as mediators for anti-neuroinflammatory responses as reported in the present work. Nevertheless, anti-inflammatory actions have been associated with GPR18 and immune cells (Honkisz-Orzechowska et al., 2024). GPR18 is the receptor for resolvin D2, and activating GPR18 which can help reduce inflammation. Finally, there are no reports of LPI-mediated interactions with GPR18. In contrast, there is a reported interaction between a synthetic derivative of LPI – 18:1 and GPR119 (Paternoster et al, 2021). While this interaction results in the release of GLP-1 which can regulate obesity and glycemic control, there has been no reported effect on anti-inflammatory actions with LPI-18:1 Taken together, these reports do not completely rule out the possibility that LPI-18:1 and other LPIs could be ligands at these other L-S receptor in DRG cultures; however, the lack of expression of GPR18 and GPR119 in the hippocampus makes this alterative explanation of LPI-mediated effects on anti-inflammatory actions less likely in the context of the present studies.

### High Content Features

A unifying observation that emerged from these studies was the *high potency* of the LPI-mediated responses observed in two primary neuronal cultures systems. LPI responses, be they either pro-inflammatory as in the singular case of LPI-20:4 or as anti-inflammatory as in the cases of LPI- 18:1, LPI: 16:0 and LPI-18:0, the mean half-maximal potencies were always sub-nanomolar. A distinguishing feature of the current work was that all measures of inflammatory markers were measured as μ2 of immunoreactive area / neuron as detected by high content fluorescent imaging. Our experimental systems were comprised of primary neurons which possess the endogenous GPR55 receptor target. The observations that LPI had actions at sub-nanomolar concentrations were likely influenced by the GPR55 target being in its natural milieu and in proximity within mitochondria (Brenneman et al., 2022) for the neuroinflammatory responses. In contrast, LPI-mediated effects have been often observed at much higher concentrations (1–10 μM) in non-neuronal cells (Martinez-Aguilar et al.,2023; Pietr et al 2009) or in GPR55-screening systems that involve overexpression strategies (Brenneman et al, 2022). As GPR55 also has reported expression in the plasma membrane (Obara et al., 2011), LPI functions at this locus may also influence the potency of the response.

Using a broad sampling in excess of 1000 neurons per value, the challenges of estimating reproducible measures from a diverse system of heterogenous neurons were made possible with an unbiased, computerized selection of pre-determined, microscopic fields in high content analyses. Further, the focus on primary neurons only for these measures obviates the potential contribution of background measures from non-neuronal cells that may or may not have similar responses. However, if this unanticipated pattern of LPI responses occurs, for example, in neurons vulnerable to human disease, the mechanism of action of GPR55 antagonists could elicit multiple neuroinflammatory outcomes. The pharmacological action produced by such a multiple-interacting system then would be critically dependent on the relative concentration and structure of these contributors for the overall neuroinflammatory response.

### Mitochondrial hypothesis: automitocrine effects

The current studies suggest that the LPI-mediated actions on the GPR55 receptor that result in the regulation of neuroinflammatory effects may constitute an “automitocrine” mechanism in which the signaling molecule (LPI) and the effector receptor (GPR55) occur within the mitochondria. Because the enzymatic acylation of LPI occurs both in microsomes and mitochondria (Keenan and Hokin, 1964), the resident mitochondrial requirement for the LPI is supported. Coupled with the previous cited evidence for mitochondrial GPR55 (Brenneman et al., 2022, both components of an automitocrine mechanism are plausible. Previous studies with melatonin and the MT1 receptor have also described such an effect in mitochondria which was given the term: “automitocrine” (Suofu et al., 2017) for its effect on blocking cytochrome c release. We would propose that the LPI/GPR55 receptor-mediated regulation of neuroinflammation provides another example of such an “automitocrine effect”. While this type of signaling is non-conventional, it opens the possibility of a new approach to our understanding of mitochondria-based regulation of neuroinflammation.

An unanticipated correlation emerged from the present study: an increase in inflammatory responses produced by LPI-20:4 was correlated with a decrease in the alamar blue viability assay. Furthermore, the decreases in inflammatory responses produced by LPI-18:1, LPI-16: 0 and LPI-18:0 were correlated with an increase in the alamar blue assay responses that were above control levels for all three structures. While it is possible that these two assay systems were not functionally related, we observed that there were potentially mitochondrial links to all of these observations. This plausible explanation has its origin in the concept that the alamar blue assay signal has proposed effects on succinate dehydrogenase (SDH), an enzyme shared by the electron transport system and the TCA cycle. As such, this assay may comprise a partial measure of mitochondrial metabolic activity (Iuchi et al., 2019). Based on this concept, a working hypothesis has emerged from these studies: increases in neuroinflammatory responses from LPI-20:4 may be correlated with decrease in mitochondrial function, as interpreted from the alamar blue assay. A decrease in SDH activity would result in an increase in succinate which could lead to an increase in pro-inflammatory outcomes (Huang et al, 2024). Conversely, a decrease in inflammatory actions as produced by LPI-18:1, LPI-18:0 and LPI-16:0 correlated with an increase in mitochondrial activity over control and increased SDH. The present studies indicated a clear dichotomy of responses among these assays that were related to LPI fatty acyl composition. The fundamental concept emerging from these studies is the importance of measuring the concentration and diversity of various LPI structures as they relate to neuroinflammatory actions in any relevant disease state.

## Figures and Tables

**Table 1:** Control values for GPR55-positive neurons on Day 12 *In vitro*: A Comparison of Control Dorsal Root Ganglion and Hippocampal Cultures

Assay ^a^	DRG Cultures		Hippocampal Cultures		DRG / HC Area Ratio	
	Cell Bodies	Neurites	Cell Bodies	Neurites	Cell Bodies	Neurites
GPR-55	64 + 6*	195 ± 13**	91 ± 4*	261 + 11**	.70	.75
NLRP3	60 ± 4*	282 ± 9**	120 ± 4*	432 ± 18**	.50	.65
IL-1b	67 ± 2*	277 ± 17	80 ± 4*	248 ± 20	.83	.65
IL-6	55 ± 3*	237 ± 13^	90 ± 3*	149 ± 15^	.71	1.59
IL-18	70 ± 4*	131 ± 11**	97 ± 7*	287 ± 11**	.72	.46
TNF-a	64 ± 8	88 ± 7 ^	68 ± 5	44 ± 3 ^	.94	2.00
Mean of All of the above	64 ± 4	201 + 11	91 ± 5	236 ± 13	.73	1.02
b-tubulin-3	367 ± 13 ^	4929 ± 230**	333 + 9 ^	5273 + 185**	1.10	.94

a Immunoreactive spot area (μ^2^)/neuron

* Significantly greater in hippocampal cultures (P < .001) than DRG cultures

** Significantly greater in hippocampal cultures (P < 0.02) than DRG cultures

^ Significantly greater in DRG cultures (P < 0.001) than hippocampal cultures
